# *Plasmodium vivax msp*-*3α* polymorphisms: analysis in the Indian subcontinent

**DOI:** 10.1186/s12936-016-1524-y

**Published:** 2016-09-23

**Authors:** Anju Verma, Hema Joshi, Vineeta Singh, Anup Anvikar, Neena Valecha

**Affiliations:** 1 Division of Plant Sciences and Bond Life Sciences Center, University of Missouri-Columbia, Columbia, MO 65211 USA; 2 National Institute of Malaria Research, Sector 8, Dwarka, Delhi, 110077 India

**Keywords:** *Plasmodium vivax*, Merozoite surface protein-3α, PCR-RFLP, Genetic variability

## Abstract

**Background:**

*Plasmodium vivax* is the most widely distributed human malaria parasite and accounts for approximately the same number of malaria cases as *Plasmodium falciparum* in India. Compared with *P. falciparum*, *P. vivax* is difficult to eradicate because of its tendency to cause relapses, which impacts treatment and control strategies. The genetic diversity of these parasites, particularly of the merozoite surface protein-3 alpha (*msp*-*3α*) gene, can be used to help develop a potential vaccine. The present study aimed to investigate the genetic diversity of *P. vivax* using the highly polymorphic antigen gene *msp*-*3α* and to assess the suitability of using this gene for population genetic studies of *P. viv*ax isolates and was carried out in 2004–06. No recent study has been reported for MSP 3*α* in the recent decade in India. Limited reports are available on the genetic diversity of the *P. vivax* population in India; hence, this report aimed to improve the understanding of the molecular epidemiology of the parasite by studying the *P. vivax msp*-*3α* (*Pvmsp*-*3α*) marker from *P. vivax* field isolates from India.

**Methods:**

Field isolates were collected from different sites distributed across eight states in India. A total of 182 blood samples were analysed by a nested polymerase chain reaction-restriction fragment length polymorphism (PCR-RFLP) technique using the *Hha*I and *Alu*I restriction enzymes to determine genetic *msp*-*3α* variation among clinical *P. vivax* isolates.

**Results:**

Based on the length variants of the PCR products of *Pvmsp*-*3α* gene, three allele sizes, Type A (1.8 kb), Type B (1.5 kb) and Type C (1.2 kb) were detected among the 182 samples. Type A PCR amplicon was more predominant (75.4 %) in the samples compared with the Type B (14.3 %) and Type C (10.0 %) polymorphisms. Among all of the samples analysed, 8.2 % were mixed infections detected by PCR alone. Restriction fragment length polymorphism (RFLP) analysis involving the restriction enzymes *Alu*I and *Hha*I generated fragment sizes that were highly polymorphic and revealed substantial diversity at the nucleotide level.

**Conclusions:**

The present study is the first extensive study in India using the *Pvmsp*-*3α* marker. The results indicated that *Pvmps*-*3α*, a polymorphic genetic marker of *P. vivax*, exhibited considerable variability in infection prevalence in field isolates from India. Additionally, the mean multiplicity of infection observed at all of the study sites indicated that *P. vivax* is highly diverse in nature in India, and *Pvmsp*-*3α* is likely an effective and promising epidemiological marker.

**Electronic supplementary material:**

The online version of this article (doi:10.1186/s12936-016-1524-y) contains supplementary material, which is available to authorized users.

## Background

*Plasmodium vivax* is the most widely spread human malaria parasite, infecting 22 million people each year outside of sub-Saharan Africa. *Plasmodium vivax* has a firm hold in South and Southeast Asia, where it accounts for 80 % of the total estimated cases from three countries: India, Indonesia and Pakistan [1]. Although *P. vivax* malaria is acute and excruciating, it is considered a benign form of tertian fever with fewer complications compared with *Plasmodium falciparum* [2]. However, recent evidence has indicated the occurrence of complicated vivax malaria cases, especially in Asia [3, 4]. The problem is further compounded by the emergence of drug resistance in vivax malaria [5–12]. The partial effectiveness of drug treatments and subsequent recurrent infections contribute to severe anaemia from vivax malaria [13].

In India, high endemicity in conjunction with the dense population contributes to the burden of vivax malaria, leading to vast social and economic consequences. Recent reports indicated equal prevalence of both *P. vivax* and *P. falciparum* in India [14]. Despite its prevalence, the lack of knowledge regarding global endemicity is a major hindrance to controlling vivax malaria [15]. Investigating the parasite population structure for genetic polymorphisms could aid in understanding the role of genetic diversity in malaria transmission [16] and is essential for the control and elimination of malaria [17].

Despite the high prevalence of *P. vivax*, few studies have investigated the genetic diversity of natural *P. vivax* populations in India. The *P. vivax* genome exhibits greater diversity compared with *P. falciparum* [18] and displays high levels of antigenic polymorphisms, which indicates the presence of sophisticated mechanisms to evade the human immune system [19]. However, recurrent infections that arise from recrudescence, re-infection or relapses make it difficult to interpret the results of clinical *P. vivax* studies that aim to determine the efficacy of treatment strategies.

Elucidating the genetic diversity of Indian *P. vivax* isolates using antigenic markers could augment the understanding of the biology and epidemiology of *P. vivax*. Additionally, understanding host genetics and environmental conditions will further aid in developing strategies for the effective control of malaria. To date, a number of single-copy antigenic genetic markers have been tested and reported for Indian isolates [20–24].

The gene of the *P. vivax* merozoite surface protein-3 alpha (*Pvmsp*-*3*α) is one of the most commonly characterized molecular markers in *P. vivax* genotyping studies. *Pvmsp*-*3*α is a potentially an important vaccine candidate expressed on the merozoite surface of the parasite and encodes a protein with three blocks of alanine-rich domain containing heptad repeats and is predicted to form α-helical coiled-coil tertiary structures [25]. Sequence polymorphism is concentrated in the central domain and can comprise of numerous point mutations and large insertions and deletions [26, 27]. These polymorphisms confer antigenic diversity in *Pvmsp*-*3*α; however, the alanine-rich heptad repeats, which are predicted to form an intramolecular coiled-coil, are conserved [28]. The *msp3* paralogs from *P. vivax* show weak similarity to *msp3* gene family members on *P. falciparum* [29]. Although both PfMSP-3 and PvMSP-3 protein contain blocks of alanine-rich heptad repeats followed by an acidic C-terminus [30], there is no supporting evidence of msp3 family of *P. falciparum* being a homologue to that of *P. vivax* [28]. In India, this polymorphic marker has been characterized in Kolkata, which is a hypo-endemic region [20], and in Chennai, which exhibits variable endemicity [21] and extensive polymorphisms in the single-copy gene and encoded protein. High genetic diversity in this locus has also been reported in South and Southeast Asian countries, including Korea [31], Nepal [32], Pakistan [33, 34], Bangladesh [35], Sri Lanka [36] and Thailand [37].

Studying the pattern of variation and distribution of *Pvmsp*-*3α* polymorphisms in different blocks of MSP-3α among isolates of South Asia and around the world will aid in understanding of the evolutionary mechanisms underlying variation patterns [38]. Studies on genetic variation in different regions with malaria have revealed numerous alleles and specific variants in different MSP-3α blocks. Additionally, the allelic forms in different blocks were observed in diverse populations worldwide [39–43]. PvMSP3α is composed of four regions which include an N-terminal signal sequence, polymorphic alanine rich repeat region as block I, less variable region as block II and an acidic C-terminus. Deletion within block I region is the basis of size polymorphism in *msp*-*3α* whereas in block II, the variations was clustered around the two structural motifs (motif I: MSELEK/LSKLEE and motif II: TAANVVKD/KEATAAKL) [26, 38, 44]. Among different populations block II was found to be relatively conserved with synonymous and non-synonymous mutations. Synonymous mutations were seen in low frequency and were population specific however, non-synonymous mutations were extensively shared among different parasite populations [38]. Restriction fragment length polymorphism (RFLP) analysis was used to determine the diversity of this gene using sequenced representative isolates. Patterns and levels of genetic diversity provide insight into population structure and aid in testing for balancing or negative selection acting on this gene.

Analysing *P. vivax* population structure is fundamental to understanding the role of genetic diversity in the transmission of malaria. Moreover, knowledge of the magnitude of the genetic polymorphisms within *P. vivax* populations is an important element for the development of strategies to effectively control malaria [45]. *Pvmsp*-*3*α is a potential antigen for vaccine development, as indicated by a study of small children in a malaria-endemic region, Papua New Guinea (PNG) [46]. Variation in the *msp*-*3α* allelic patterns of *P. vivax* in India provides fundamental knowledge for inferring *P. vivax* population structure and therefore information that can be used to help design MSP-3-based malaria vaccines.

In this paper the extent of allelic and sequence diversity in *Pvmsp*-*3α* in field isolates collected from different geographical regions of India with varied malaria transmission patterns was investigated. To accomplish this, isolates from different endemic areas were collected and amplified by nested polymerase chain reaction (PCR) and analysed using PCR-RFLP analysis.

## Methods

### Study sites

**Fig. 1:**
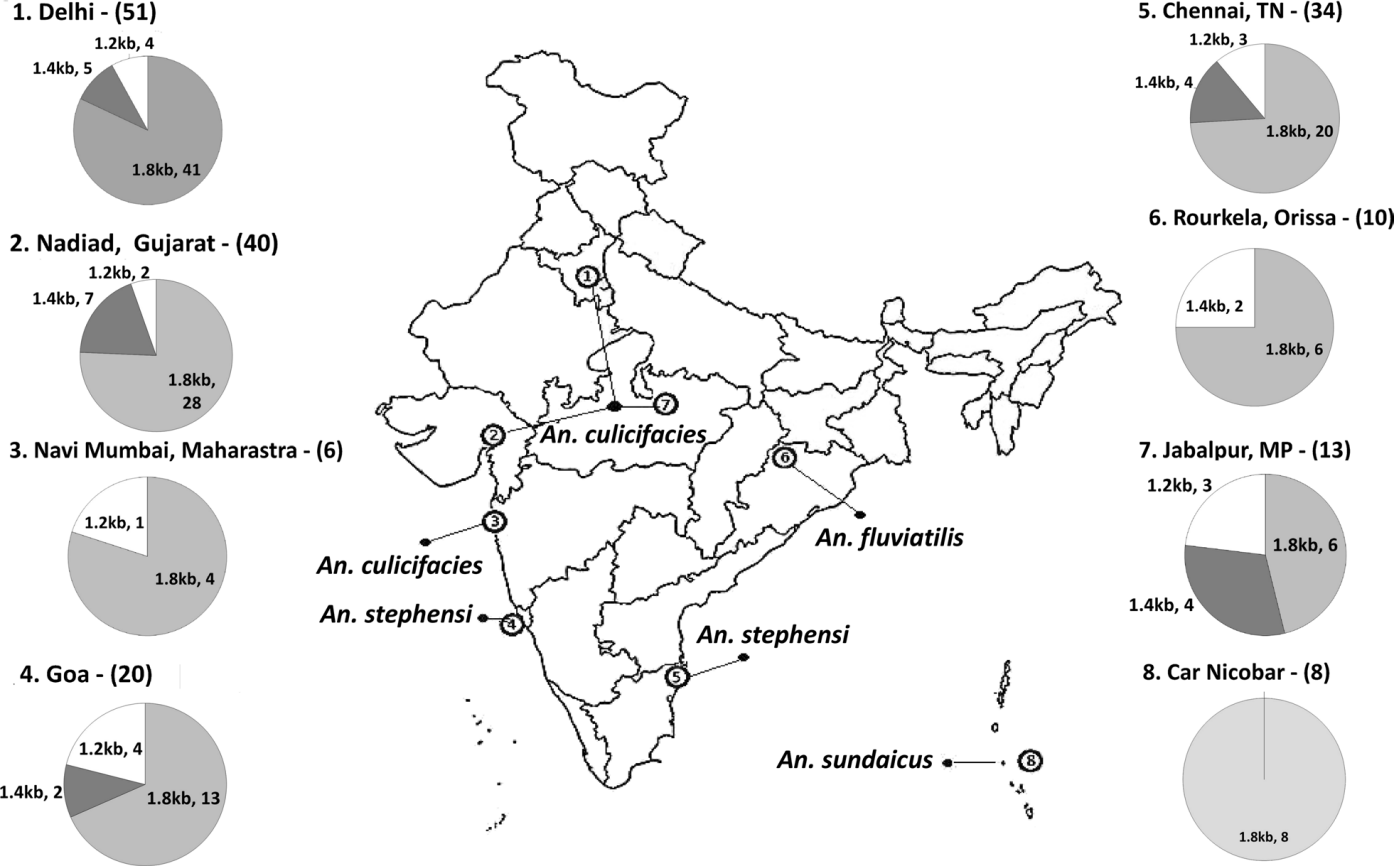
*Map of India* indicating the study sites and *graphic representation* of the geographical distribution of the frequencies of the *Pvmsp*-*3α* haplotypes in isolates from the Indian sub-continent

Blood samples were collected between 2000 and 2004 during spot surveys from patients who attended malaria diagnosis clinics at the Malaria Research Centre headquarters in Delhi or within field units. A total of 182 finger-prick blood samples were obtained from individuals from eight different geographical regions of the Indian sub-continent, including coastal, mainland and island regions (Fig. 1). In the northern region of the country, samples were collected from Delhi, Panna (Madhya Pradesh) and Nadiad (Gujarat). The *Anopheles culicifacies* vector is the primary vector in this region, and transmission is mainly in the post-monsoon months (July–October). The blood samples were collected during malaria outbreaks in all study sites except Delhi. Samples were collected from rural settings in Nadiad, and tribal dominant forest ecotypes in Panna. Sundergarh (Orissa), a tribal-dominated area under the influence of the highly anthropophagic vector *Anopheles fluviatilis*, is a hyper-endemic district within the state of Orissa, which has reported more than 90 % of known falciparum malaria cases. Navi Mumbai (Maharashtra), Goa and Chennai (Tamil Nadu) are all coastal regions in which *P. vivax* is dominant. In Chennai, which is located on the eastern coast of India, *Anopheles stephensi* is a major vector in urban areas, whereas *An. stephensi* and *An. culicifacies* are responsible for the transmission of malaria in Goa and Navi Mumbai (on the western coast). On Car Nicobar Island (Bay of Bengal), *Anopheles sundaicus* is the primary endemic vector of malaria, but reports of perennial transmissions by *P. vivax* and *P. falciparum* occur throughout the year.

The blood smears were stained with Jaswant Singh Bhattacharya stain [47] and examined under 1000× magnification. The samples collected were spotted onto autoclaved Whatman filter paper (3 mm) strips, and dried blood spots were stored at 4 °C in plastic bags prior to the extraction of parasite DNA. The study was approved by the ethics committee of the National Institute of Malaria Research. Informed consent was obtained from all adult participants and from the legal guardians of minors.

### Genomic DNA isolation

Extraction of parasite genomic DNA from individual dried blood spots on Whatman filter paper strips was carried out using the QiAamp DNA extraction mini kit (Qiagen, Hilden, Germany) based on the manufacturer’s instructions. Extracted samples were re-dissolved in 100 μl triple-distilled autoclaved water and used as a template for PCR amplification or stored at −20 °C until use.

### *Pvmsp*-*3α* PCR analysis

*Plasmodium vivax* genotyping was carried out using a nested PCR assay. The protocols used for the PCR assays were reported by Bruce et al. [48]. A 5-µl aliquot of each PCR product was visualized on a 1 % agarose gel (Boehringer Mannheim, Indianapolis, USA). Only the samples showing single a PCR fragment were considered for RFLP analysis.

### RFLP analysis

Characterization of the allelic patterns of the 167 PCR single clone isolates obtained was performed using the *Alu*I and *Hha*I restriction enzymes based on the methods reported by Bruce et al. [48]. To determine the level of *Pvmsp*-*3α* polymorphism, RFLP analysis was carried out using the *Hha*I and *Alu*I restriction enzymes (NEB, Inc, Beverly, MA, USA) in a 10-µL reaction volume. A 5-µl aliquot of each PCR product was digested individually with the restriction enzymes along with BSA in the buffer supplied with the enzymes at 37 °C for 4 h. The restriction products were then visualized on a 3.0 % agarose gel.

### DNA sequencing

Twenty isolates that represented all three of the variants were sequenced and subjected to restriction pattern analysis. These included six samples each of Type B and C and 8 samples of Type A. Conserved primers were designed for nested PCR amplifications to target the block-I, beta, and block-II regions that covered the entire alanine-rich region of *Pvmsp*-*3*α [43], which corresponded to nucleotide positions 205 bp to 2,100 bp (69–700 aa) of the Belem strain [27]. The nested PCR products that were sequenced were subjected to in silico simulation using a restriction mapper [49] to map the restriction sites and generate fragment sizes. The amplified PCR products from a limited number of isolates from each area were sequenced using the BigDye^®^ terminator cycle sequencing Kit (Applied Biosystems, Foster City, CA, USA) and the ABI Prism^®^ 310 Genetic Analyzer (Applied Biosystems). Curation was performed using the ABI Prism^®^ sequence analysis software (version 3.4, Applied Biosystems). Six internal primers were designed using the Belem strain as a standard to cover the 1.8-kb length and sequenced in both directions for accuracy. The following primers were used: sense strand primers, including *Pvmsp3α*_F1, 5′-CCGGAAATGCCAACAA-3′; *Pvmsp3α*_F2, 5′-CAAAGGCGGAAGTGCTGAAC-3′;*Pvmsp3α*_F3: 5′-GAGAAAAAGCAGAAAA.

GGTAGGAG-3′; and anti-sense primers, including *Pvmsp3α*_R1, 5′-CCGCCTTTTCCTTTGCCTTAATTTG-3′; *Pvmsp3α*_R2, 5′-GCCTTTTCTG CCTGCTTCTTA-3′; and *Pvmsp3α*_R3, 5′-TGCTTTATTTTTAGTTTCTTGTGG-3′. In addition, two nested primers, N1 and N2, were used as described by Bruce et al. [48].

### *Pvmsp*-*3α* sequence and phylogenetic analysis

The resulting nucleotide sequences, amino acid sequences that were subsequently deduced from the nucleotide sequences, and multiple sequence alignments were analysed using MEGA 6.06 [50, 51], and the aligned sequences were analysed for phylogenetic inference. To determine the details of *Pvmsp*-*3α* sequence diversity, PCR products of 20 samples were aligned with the Belem I reference gene (AF093584) and Salvador I sequence (AAO20890). Restriction site analysis of the *Pvmsp*-*3α* sequences from various geographical regions was carried out using restriction-mapping software [49]. The Indian isolates were also compared with 80 published *Pvmsp*-*3*α sequences from isolates from different geographic locations including India (KC935446, AF491957, HQ328853-55), Bangladesh (AF491951, AAO20882), Sri Lanka (AF491961 and GU175269-72), Pakistan (AY266090), Thailand (AY833010-26), North Korea (AF491958), South Korea (EF204144-63), Myanmar (EU430576-86), PNG (AF491950, AF491959), Borneo AY118174), Venezuela (KC935422-26), Ecuador (AF491952), Belem (AF093584), and Sal_I (AAO20890). Evolutionary relationships of the Indian isolates and published *Pvmsp*-*3α* sequences were determined based on the construction of a phylogenetic tree of nucleotide sequences using the reference strains and the maximum likelihood method, which is based on the Kimura two-parameter distance estimation method [51] using Mega 6.06. To obtain a confidence value for the aligned sequence dataset, bootstrap analysis of 1000 replications was conducted using Mega 6.06.

### Ethical clearance

Study has clearance of Ethics committee of National Institute of Malaria Research.

## Results

### *Pvmsp3*-*α* allelic frequency

A total of 182 *P. vivax*-positive samples confirmed by microscopy were further investigated by PCR. *Pvmsp3*-*α* exhibited a high level of genetic diversity among the field isolates collected from the different study areas. PCR analyses of the isolates from the eight different sites in India revealed the presence of three variants of different lengths: Type A (1.8 kb), Type B (1.4 kb) and Type C (1.2 kb), which were observed in isolates from across the study sites, and Type A variant among the isolates was the most prevalent (Table 1). The geographical distribution of the *Pvmsp*-*3α* variant frequencies of the isolates from the Indian sub-continent is shown in Fig. 1. The frequencies of the different types of alleles among the field isolates were 68.8 % (126/182) for the Type A, 13.1 % (24/182) for the Type B, and 9.28 % (17/182) for the Type C (Table 1).

**Table 1:** Distribution of the three *Pvmsp*-*3α* haplotypes among the Indian states based on PCR and RFLP analysis

State	Total samples	PCR based single clones			PCR based multiple clones	PCR-RFLP based single clones			PCR-RFLP based MOI
		Type A	Type B	Type C		Type A	Type B	Type C	
Chennai, TN	34	20	4	3	7	19	4	3	1
Delhi	51	41	5	4	1	32	3	3	12
Goa	20	13	2	4	1	12	2	4	1
Car Nicobar	8	8	0	0	0	7	0	0	1
Navi Mumbai, Maharashtra	6	4	0	1	1	4	0	1	0
Nadiad, Gujarat	40	28	7	2	3	23	7	2	5
Rourkela, Orissa	10	6	2	0	2	5	2	0	1
Jabalpur, MP	13	6	4	3	0	5	2	1	5
Total	182	126	24	17	15	107	20	14	26

### Multiple clones

Mixed-genotype infections or multiplicity of infections (MOI) were detected based on the size of the polymorphism. Samples were designated as mixed infections when the PCR analysis resulted in two or more products of different size or when the RFLP analysis of a single PCR band exceeded the size of the uncut PCR band. Among all of the samples analysed, 8.2 % (15/182) were mixed *Pvmsp*-*3α* infections detected by PCR alone. RFLP analysis was also carried out to detect mixed infections. The total number of multiple clones detected by RFLP and PCR was 22.4 % (41/182). However, RFLP analyses for detecting multiple clones is challenging and needs to be dealt with caution as incomplete digestion may result in pseudo bands. The highest number of multiple infections detected by PCR was in the Chennai (20.5 %) and Rourkela (20 %) samples (7/34 and 2/10, respectively) (Table 1).

### *Pvmsp*-*3α* PCR-RFLP analysis

Isolates that contained a single allele, as determined by PCR, were further analysed by PCR-RFLP, but mixed infection cases were excluded. The *Hha*I restriction enzyme showed clear patterns in 139 out of the 167 samples digested (Additional file 1). In total, 26/167 samples (15.5 %) exhibited fragment sizes greater than the PCR fragment and inconsistent fragment patterns. These fragments were possibly multiple-clone infections and not included in the analysis. The PCR-RFLP patterns from 141 samples revealed 56 different alleles using the *Hha*I restriction enzyme (Additional file 1). The most prevalent alleles were H1 for the Type C haplotype, H2 for the Type B haplotype, and H3, H9, H17, H25 and H33 for the Type A haplotype, which were present in approximately 43.5 % (61/140) of the isolates. (Additional file 1: Figure S1, Additional file 2: Table S1, Additional file 3: Table S2). The genotypic combinations of the *Alu*I and *Hha*I enzymes were most diverse in the Type A variant, followed by the 1.4-kb variant.

### Sequence data

#### Indian isolates

Sequencing of 20 isolates revealed that, among the Indian isolates, the length of the Type A variant ranged from 1883 to 1938 bp, whereas the length of the Type B variant ranged from 1452 to 1461 bp and the length of the Type C variant ranged from 1128 to 1137 bp. Digestion of the three variants with *Hha*I revealed that a 1-kb fragment was conserved in almost all three of the variants (Additional file 1). This band was observed to be slightly polymorphic on gels following electrophoresis, and sequencing data revealed that the fragment size ranged between 929 and 989 bp. Very few restriction patterns obtained with the *Alu*I enzyme were identical or even similar for closely situated geographical regions, which indicates that the higher resolution of the bands obtained by actual restriction digestion resulted in numerous alleles observed among the Indian isolates.

**Fig. 2:**
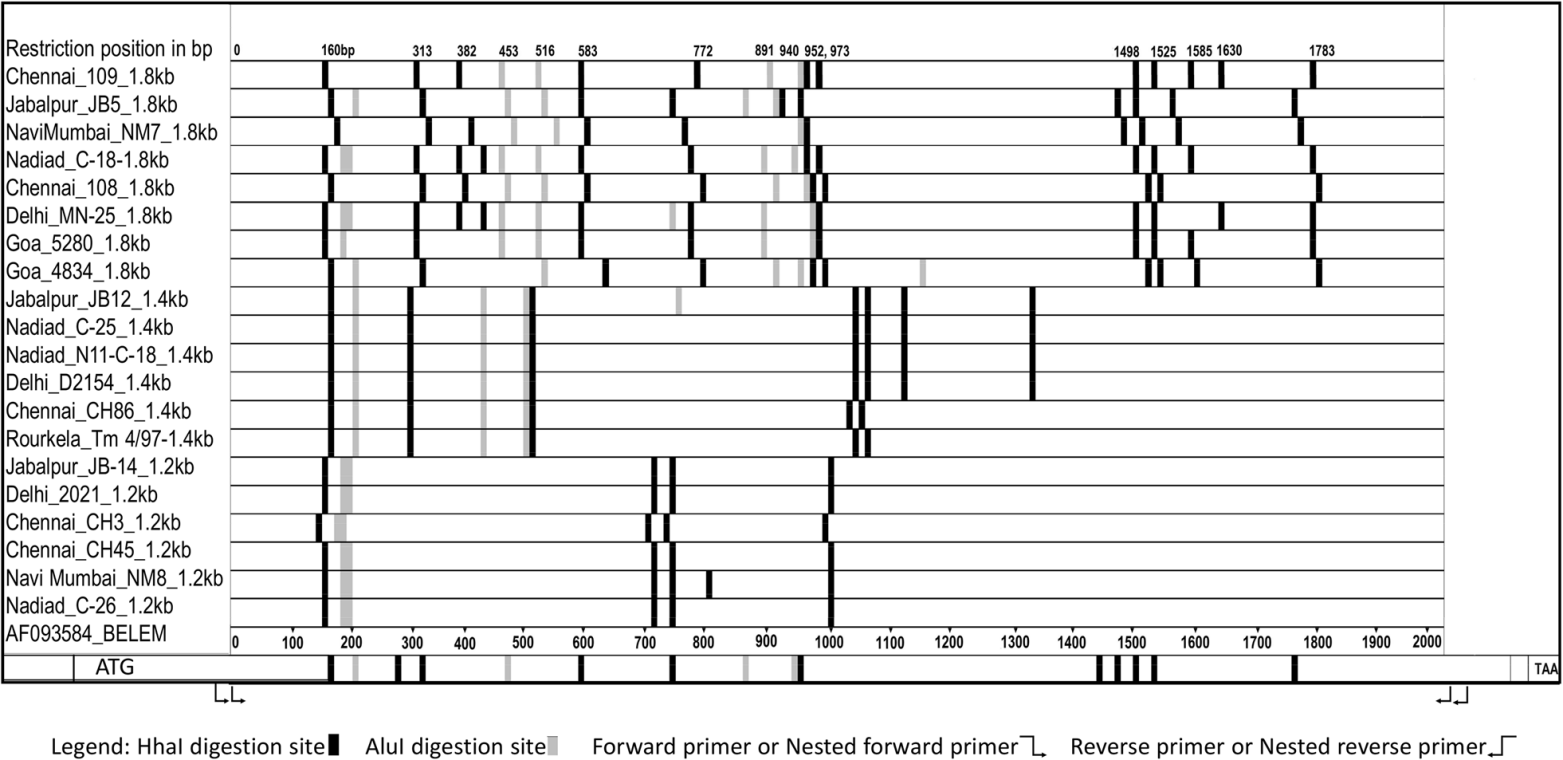
*Alu*I and *Hha*I restriction sites of *Pvmsp*-*3α* gene using in silico digestion of 20 isolates from Indian sub-continent (drawn according to scale)

In silico digestion of the nested PCR products with *Alu*I and *Hha*I resulted in *msp*-*3α* sequences being cut into a range of four to 13 bands for *Alu*I and three to eight bands for *Hha*I. The size and combinations of the virtually digested bands and sequence diversity at and between cut sites allowed us to distinguish eight *Alu*I and *Hha*I haplotypes among each of the eight sequences in the Type A (Fig. 2). For the Type B variant, two *Alu*I and three *Hha*I haplotypes were observed, and for the Type C variant, two haplotypes each for *Alu*I and *Hha*I were identified among the sequences.

#### *Pvmsp*-*3α* sequence comparison

**Fig. 3:**
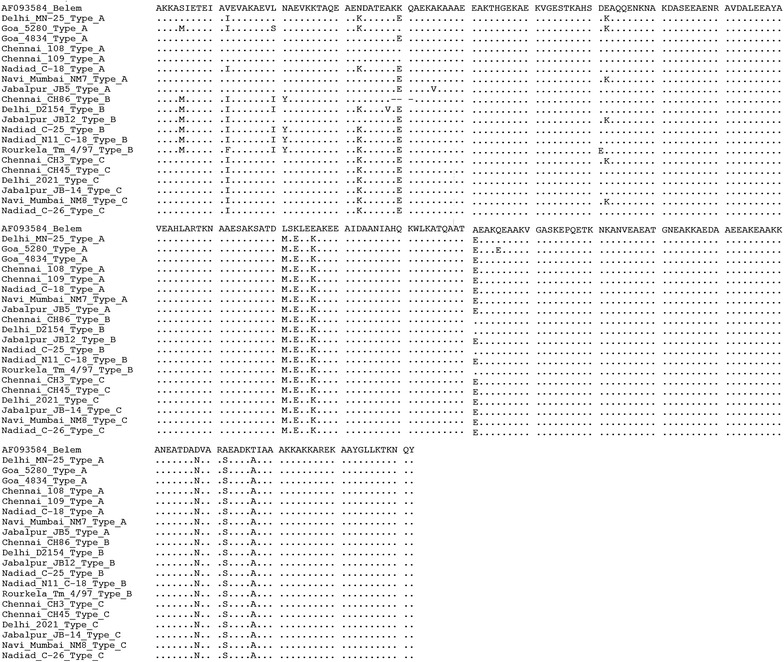
Alignment of *Pvmsp*-*3*α block II translated sequences showing polymorphism among Indian isolates. *Dots* and *dashes* represent identical residues and deletions, respectively

Type A and Type B PCR fragments contained 480 and 789 bp deletions, respectively, in block-I, whereas block-II was relatively conserved in the 20 samples (Fig. 3). *Pvmsp*-*3α* block-II in the 20 samples had 28 single nucleotide polymorphisms and sequence data analysis revealed 83.59–99.47 % sequence identity among themselves, and the average identity of the Indian isolates compared with isolates around the world was above 74 %. All of the sequenced *P. vivax* isolates examined in this study showed greater identity (range, 95–99 %) to the previously sequenced isolates from India (GenBank accession numbers HQ328853, HQ328854, HQ328855) than to sequences from South Asian or other regions worldwide. Figure 3 shows alignment of block-II amino acid sequences of the *Pvmsp*-*3*α protein from the 20 different Indian isolates and the original sequence from the Belem strain of *P. vivax.* The most striking feature of the alignment of the different *msp*-*3α* blocks was the presence of numerous polymorphic sites.

**Fig. 4:**
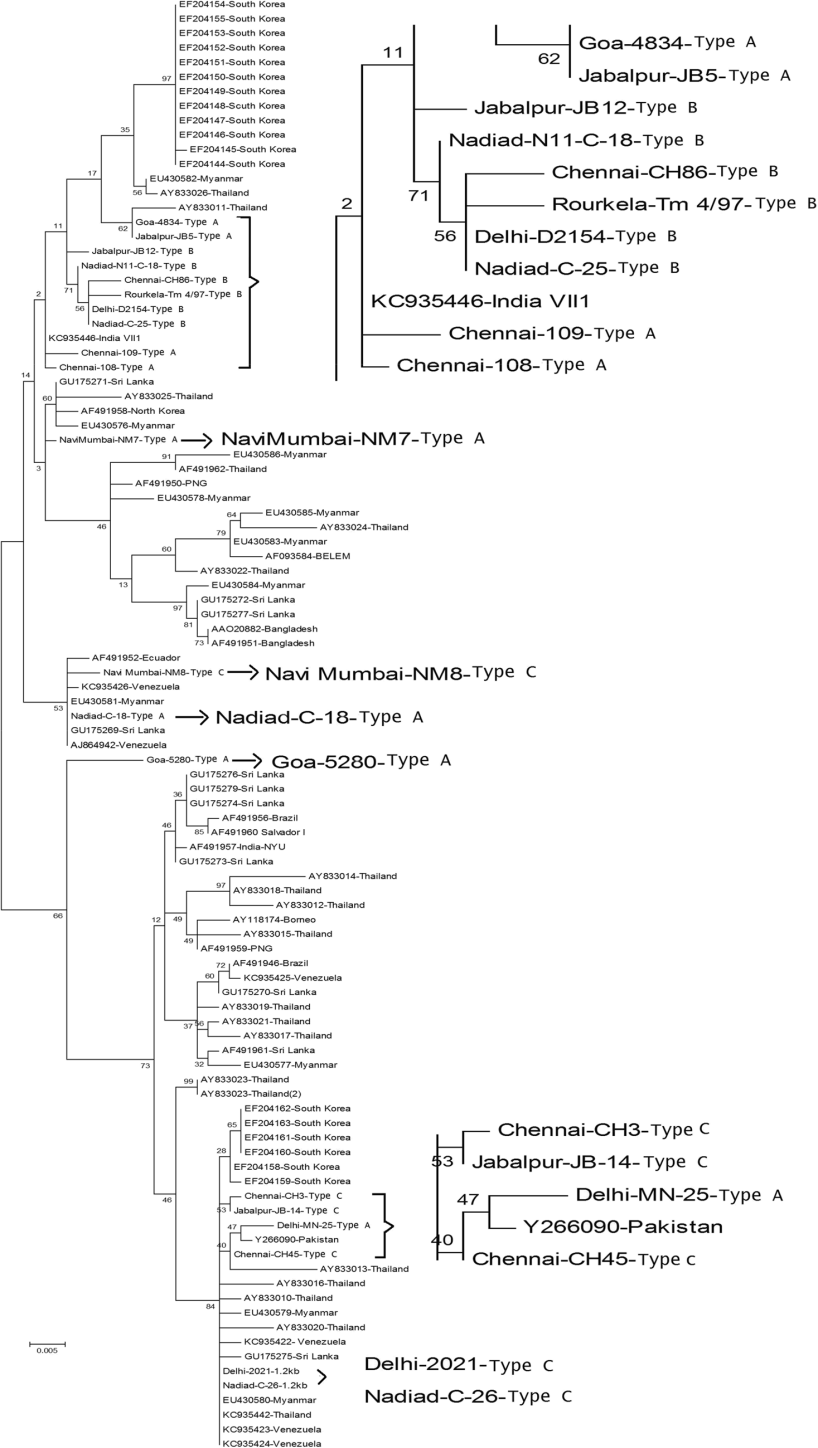
Phylogenetic relationships among the 20 Indian isolates sequenced and previously known Indian isolates of the *Plasmodium vivax* merozoite surface protein-3α (*Pvmsp*-*3α*) gene of the Indian isolates were compared to Belem strain (AF093584) was conducted using MEGA 6.0 using the reference strains and the maximum likelihood method, which is based on the Kimura two-parameter distance. Phylogenetic analysis of the *Plasmodium vivax msp-3α* gene. The phylogenetic tree for the 100 isolates, including 20 isolates from the present study of *Pvmsp-3*α, was constructed with maximum likelihood method based on the nucleotide sequences of the Indian isolates using the MEGA6 program. Numbers on the branches indicate bootstrap proportions (1000 replicates)

A phylogenetic tree of the *Pvmsp*-*3α* sequences was constructed using the maximum likelihood method based on the nucleotide sequences of the Indian isolates in this study and isolates from South Asia, Southeast Asia and isolates around the world (Fig. 4). Distances were calculated using the bootstrap method with nucleotides 207–2100 bp of the Belem strain (GenBank accession number: AF093584). The Indian isolates from the study were compared to Belem and Salvador I strains. Among the Indian isolates, clustering of Type B was seen while Type A and C were more interspersed among the world isolates (Fig. 4). The three variants based on PCR length polymorphism mainly fell into separate groups, which indicates that a similar genotype prevailed in distinct geographic areas as a result of similar selection pressure. The Indian isolates compared to 80 published gene bank *Pvmsp*-*3α* sequences from different geographic locations, mainly from South Asia, Southeast Asia and a few from other geographic locations worldwide. Comparison of the Indian isolates to other isolates revealed clustering into two distinct groups for the Belem and Salvador I type. The analysis revealed almost equal distribution of the Indian isolates in both the clusters. Some of the *P. vivax* isolates examined in this study showed 100 % identity with strains of South Korea isolates (Gene bank accession numbers: EF204158 and EF204163) and greater than 99 % similarity to isolates from Thailand, South Korea and Myanmar. The remaining isolates were new alleles identified in this study.

## Discussion

Malaria remains the most devastating global human parasitic infection, and in 2015, about 214 million malaria cases and an estimated 438,000 malaria deaths were reported [52]. Additionally, the incidence of malaria in India accounted for 58 % of cases in the South Asian region [1]. *P. vivax* is the most geographically widespread human malaria parasite, and it annually accounting for 70–80 million clinical cases throughout tropical and sub-tropical regions worldwide. In India, *P. vivax* causes approximately 42–45 % of all malaria infections [53].

To develop and evaluate suitable novel control strategies against this parasite, it is important to know the extent of the polymorphisms that exist in the population. Despite more than a century of efforts to eradicate or control malaria, parasite diversity and the genetic variability of the *Plasmodium* parasites has made it difficult to eradicate this disease. The variability in the polymorphic markers impairs the effectiveness of antibody repertoires generated during previous infections and also hinders development and testing of new drugs and malaria vaccines [54]. This study was carried out with the intention to further understanding of the genetic structure and estimate the extent of the diversity that exists in *Pvmsp*-*3α* among the isolates of the Indian subcontinent.

Observations in the present study revealed that *Pvmsp*-*3α* was a highly diverse and polymorphic marker among the field isolates in India. Analysis of 182 field isolates collected from eight different locations showed different epidemiological patterns, which indicated extensive and diverse variation. A high degree of allelic diversity previously reported by Kim et al. [20] among the isolates of Kolkata (India), an area with low transmission of malaria, supports the highly diverse and polymorphic nature of *Pvmsp*-*3α* among Indian isolates. It is interesting to note that there was no significant difference in the observed level of diversity among the isolates of low and high endemic regions of the Indian sub-continent. Similar observations of high diversity among isolates of low-endemic areas in Thailand were made by Cui et al. [45]. It is suspected that either highly virulent isolates are not present despite high diversity or the malaria control measures in such regions are more effective for restricting the incidence to a low level.

All three of the *Pvmsp*-*3α* PCR variants (Type A, B and C) observed worldwide were present among the field isolates, although with varied frequencies in the different geographical regions. The largest size variant (Type A) occurred at the highest frequency in all samples of natural *P. vivax* populations in India, and the frequencies observed were similar to the previously reported global frequencies. The difference in the proportion of the Type A and C variants in the population might have been due to the deletions found in the smaller length variants, which might have caused a loss of fitness for the parasites that carried the variants [26]. However, these genotypes may occur in the population to balance any fitness cost associated with large deletions within *Pvmsp*-*3α* [55]. Block-I exhibits maximum sequence variability in this region, acts as a placeholder to mimic the larger length variant [56] and also confers a selective advantage to the parasite, i.e., evasion of preexisting immune responses. The observation that block-II exhibits only slight variation in size and very little polymorphism that is limited to certain portions indicates that it plays an important role in the formation of the protein structure. The Type B and Type C variants exhibited minimal sequence diversity and differed from each other in the nucleotide sequences that form the beta helix turn.

Analysis of the isolates from eight regions of the Indian sub-continent indicated that diversity was not linked to geographical region and a high level of diversity was observed within the same region. New alleles identified in the Indian isolates demonstrated high variability among the field isolates. The high allelic diversity of *Pvmsp*-*3α* has also been reported in other regions of the globe, including South Asia, Southeast Asia, PNG [46] and Latin America [55], including Peru [57] and Colombia [58]. Some of the identified alleles are consistent with those from earlier reports, which indicate global distribution of certain parasite genotypes [59]. Based on msp-3α data, the existence of a common allelic composition in different parts of the globe indicates the presence of a single random-mating population of *P. vivax* across the globe with no geographical sub-structuring. However, this may not be true as new molecular approaches such as microsatellite and mitochondrial studies have been used to assess *P*. *vivax* quantifying parasite diversity and population structure [16, 60–62]. *Hha*I digestion revealed 56 alleles and H1 allele was most prominent in the Indian sub-continent and was seen in the Type C variant among the Indian isolates. H1 allele in these variants has been reported from other regions of the world, including those from India [21, 63, 64].

Simultaneous infection of a host by more than one strain of the same parasite is common in malaria and is partially correlated with transmission intensity levels. For *P. vivax* malaria, the estimated proportion of mixed-strain infections in PNG is 65 % [65], compared with 43 % reported in India [66]. In recent studies in the Kolkata region of India, 10.6 % of recorded cases were multiple infections [20]. In the present study, 8.2 % of cases were multiple infections based on PCR detection and 22 % based on PCR-RFLP of *Pvmsp*-*3α* sequences. RFLP analyses for detecting multiplicity of infection may not be completely reliable as incomplete digestion lead to spurious results. Restriction with multiple enzymes may be helpful in detecting multiple clones by RFLP. Further, microsatellite (MS) markers and SNP’s are reliable and important tools for studying multiplicity of infection (MOI) of malaria parasite infections [28, 67, 68].

Several studies reported extensive microsatellite diversity and high multiplicity of infection in *P*. *vivax* in regions of moderate endemicity [61]. The increased multiplicity of *P. vivax* infection is attributable to the biological features of the *P. vivax* parasite, such as early gametocytogenesis and relapse [35]. Moreover, the multiplicity of infections is likely to facilitate genetic recombination of parasites, and the generation of novel strains [45].

The observed high level of *Pvmsp*-*3α* diversity among the field isolates was further supported by sequencing data, which revealed that the different genotypes of the sequenced isolates were found throughout the different geographical areas. In particular, the most common variant (Type A) displayed the maximum diversity, which was expected because of the presence of an intact block-IB in this variant, which is the most variable. However, in the smaller length variants (Type B and C), block-IB was either completely or partially deleted, thereby reducing the diversity.

Twenty samples were sequenced, and the restriction sites were analysed. No two resulting *Alu*I or *Hha*I restriction banding patterns were similar in the Type A haplotype, which indicated the extent of the variability. Most of the isolates sequenced revealed new alleles. Based on the restriction site analysis of the sequenced isolates, it can be concluded that each of the Type A haplotypes isolated was a different allele. However, when restriction sites of the sequenced Type B haplotype (identical pattern on the gel) were analysed, the restriction pattern differed slightly in terms of band size. However, the Type C fragment restriction pattern analysis of the sequenced fragment revealed a similar pattern, thus indicating reduced variability in the smaller types. The in silico restriction site analysis of the published *Pvmsp*-*3α* sequences from various geographical regions was carried out using restriction mapping software. The virtual restriction patterns obtained with either the *Hha*I or *Alu*I enzymes were not analogous to isolates from a single region or close geographical regions, which indicated that higher resolution of the bands obtained by actual restriction mapping could result in numerous alleles, as was obtained from the Indian isolates. This finding signifies the importance of sequence data as a source for estimating the exact variability and its extent among isolates from other areas globally. As reported earlier, higher levels of sequence polymorphism were observed in the region closer to the central alanine-rich domain [28, 40, 48].

*Pvmps*-*3α* is an essential polymorphic marker for studying the population structure of *P. vivax*. Numerous studies from various regions have been carried out to identify the number of *Pvmsp*-*3α* alleles using the PCR-RFLP method [20, 21, 32–37, 39–43]. Several investigators have advocated for this method as a means of identifying the variations available in this allele. However, based on the in silico digestion, *Alu*I results in complicated patterns may not be a good enzyme for PCR-RFLP purpose [28]. Virtual digestion of *Pvmsp*-*3*α sequences revealed greater variability in terms of the fragment sizes obtained, which was not resolved easily on a gel and could result in reaching a biased conclusion regarding the number of alleles. Moreover, the results may vary depending on the electrophoresis conditions. As proposed by Rice et al. [28], sequencing may be a better option to determine the sequence-level genetic diversity of the *pvmsp*-*3α* gene. Therefore, sequencing analysis is advocated for analysing the extent of the variability generated by the parasite to evade the host immune system and impart a survival advantage to the parasite [28, 69].

PvMSP-3α is a potentially important vaccine candidate expressed on the surface of the merozoite in the parasite [70]. Dual role for PvMSP-3α in both the immunity and pathogenesis of malaria has been suggested [71]. PvMSP-3α is known to elicit a pronounced antibody response against clinical malaria infections reported from PNG [46]. Additionally, it has been established that naturally acquired antibodies against the C-terminal block-II of PvMSP3α are associated with protection from symptomatic vivax malaria [46].

There is limited information available regarding PvMSP3α sequences from India. Sequence polymorphisms in Indian isolates were mainly clustered at the 5′ regions of the marker, as was seen in other isolates worldwide. However, block-II (residues 434–687aa of Belem strain), the region of the alanine-rich core displays less variability. In an earlier study phylogenic analysis based on sequence variation of 237 world sequences of PvMSP-3α block II region resulted in three robust clusters suggesting extensive gene flow between populations. However these clusters did not reveal any geographical structure. The phylogenic grouping was influenced by sequence variations of two motif (motif I: MSELEK/LSKLEE and motif II: TAANVVKD/KEATAAKL) which suggested selective pressure on these motifs [38].

In the Belem strain the motifs I and II correspond to position 529–534aa and 576–583aa respectively [26]. Among the Indian isolates, motif I was represented by MSELEK sequence in all the twenty isolates, while motif II exhibited both the sequences at different frequencies. One of the Indian isolates however represented a recombinant type of motif with an amino acid sequence of TEANVAKL, which was seen in the Goan isolate.

Comparison with published nucleotide and protein sequences in NCBI and PlasmaDB using phylogenetic trees of *Pvmsp*-*3*α revealed that most subtypes were new alleles. Two distinct clusters, one which included Belem type and the other Salvador I type were formed when the Indian isolates were compared with other isolates worldwide. Genotypes from different geographical areas were distributed in both clusters, which revealed no convincing evidence of geographical grouping based on this marker.

## Conclusions

The present study indicates a high degree of genetic diversity in clinical *Pvmsp*-*3α* isolates and is remarkably diverse, even within limited geographical areas of the Indian subcontinent. The high prevalence of mixed-clone infections revealed by this marker in the Indian sub-continent likely resulted from a high transmission rate and tropical climate, which offers a suitable environment for vector breeding. The extensive allelic diversity demonstrates *Pvmsp*-*3α* to be a very useful molecular marker for distinguishing field isolates. However, further investigation is required to elucidate the diversity and expression of the C-terminal conserved region in field isolates, which will help reveal the potential of this gene as a vaccine candidate.

## Additional files

10.1186/s12936-016-1524-y 1 (A) Polymerase chain reaction amplified fragments of Pvmsp-3α gene from P. vivax field isolates. Digestion pattern using Restriction fragment length polymorphism of *Pvmsp-3α* using AluI (B) and HhaI (C) restriction enzymes. 1kb and 100bp DNA markers.
10.1186/s12936-016-1524-y *Pvmsp-3α* RFLP pattern (Alu 1 and Hha1 restriction enzymes) and genotyping among *P. vivax* isolates collected from different cities in India.
10.1186/s12936-016-1524-y *Plasmodium vivax* Alu and HhaI MSP3a alleles and their frequencies in the Indian subcontinent.
