# Supplementary material for: Plasmodium vivax msp-3α polymorphisms: analysis in the Indian subcontinent
Source: Malar J. 2016 Sep 23;15:492. doi: 10.1186/s12936-016-1524-y (PMC5035448; doi:10.1186/s12936-016-1524-y)

Additional figure 1. (A) Polymerase chain reaction amplified fragments of *Pvmsp-3*α gene from *P. vivax* field isolates. Digestion pattern using Restriction fragment length polymorphism of *Pvmsp-3*α using AluI (B) and *HhaI (C)* restriction enzymes. 1Kb and 100bp DNA markers.

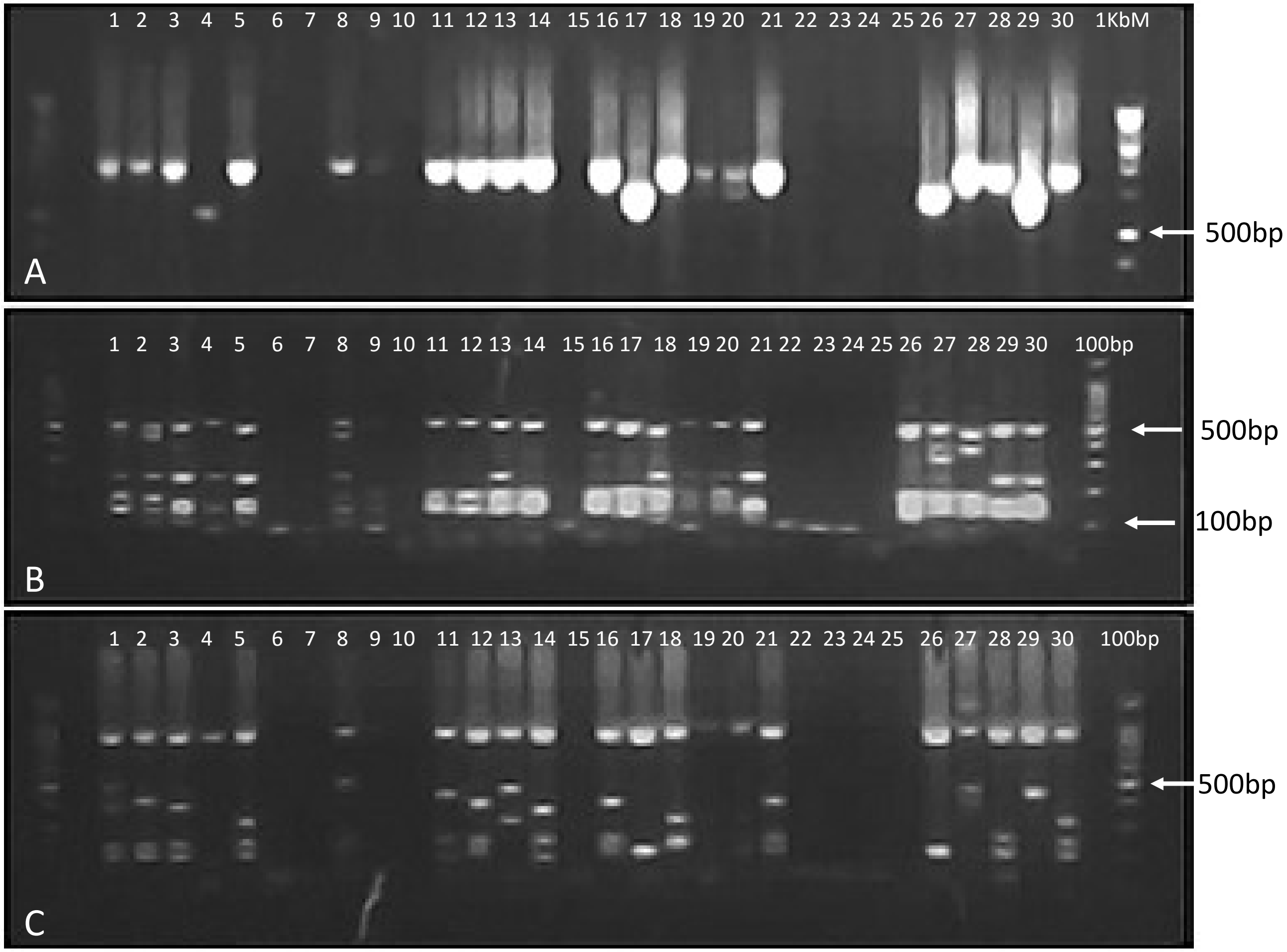

Supplement: Supplementary file 1 — 10.1186/s12936-016-1524-y 1 (A) Polymerase chain reaction amplified fragments of Pvmsp-3α gene from P. vivax field isolates. Digestion pattern using Restriction fragment length polymorphism of Pvmsp-3α using AluI (B) and HhaI (C) restriction enzymes. 1kb and 100bp DNA markers. [file 12936_2016_1524_MOESM1_ESM.pdf]
